# Adapalene‐Associated Cutaneous Adverse Events: A Disproportionality Analysis of the FAERS Database

**DOI:** 10.1111/jocd.71207

**Published:** 2026-09-24

**Authors:** Zhongping Cao, Chenyu Zhao

**Affiliations:** ^1^ Department of China Medical University‐The Queen's University of Belfast Joint College, School of Pharmacy China Medical University Shenyang China; ^2^ School of Pharmacy Queen's University Belfast Belfast UK

**Keywords:** adapalene, cutaneous adverse events, pharmacovigilance, skin burning sensation

To the Editor,

Adapalene is a third‐generation topical retinoid which is highly effective, generally well‐tolerated first‐line topical treatment for acne [1, 2]. Its expected local effects, including dryness, erythema, burning, scaling, and irritation, are familiar to dermatologists. However, spontaneous‐reporting data may capture a wider spectrum of cutaneous terms, including events that overlap with the treated disease or are not explicitly represented in product labeling. We therefore characterized adapalene‐associated cutaneous reporting patterns and distinguished expected reactions from indication‐related terms and potentially under‐recognized signals.

FAERS quarterly files from Q1 2004 through Q4 2025 were analyzed. Reports were included when adapalene was designated the primary suspect drug; ingredient‐level standardization included adapalene‐containing fixed combinations. Duplicate case versions were removed by retaining the record with the latest FDA receipt date for each CASEID and, when dates were tied, the highest PRIMARYID (Figure 1). Events were coded at the system organ class (SOC) and preferred term (PT) levels using MedDRA version 26.1. Four complementary disproportionality approaches were applied: reporting odds ratio (ROR), proportional reporting ratio (PRR), Bayesian confidence propagation neural network information component (IC), and multi‐item gamma Poisson shrinker empirical Bayes geometric mean (EBGM). A drug‐PT pair was considered a signal only when all prespecified criteria were met: at least three reports, lower 95% confidence bounds for ROR and PRR greater than 1, IC025 greater than 0, and EBGM05 greater than 2 (Table S1). Counts refer to drug‐event records rather than unique patients; one case may contribute multiple PTs.

**FIGURE 1:**
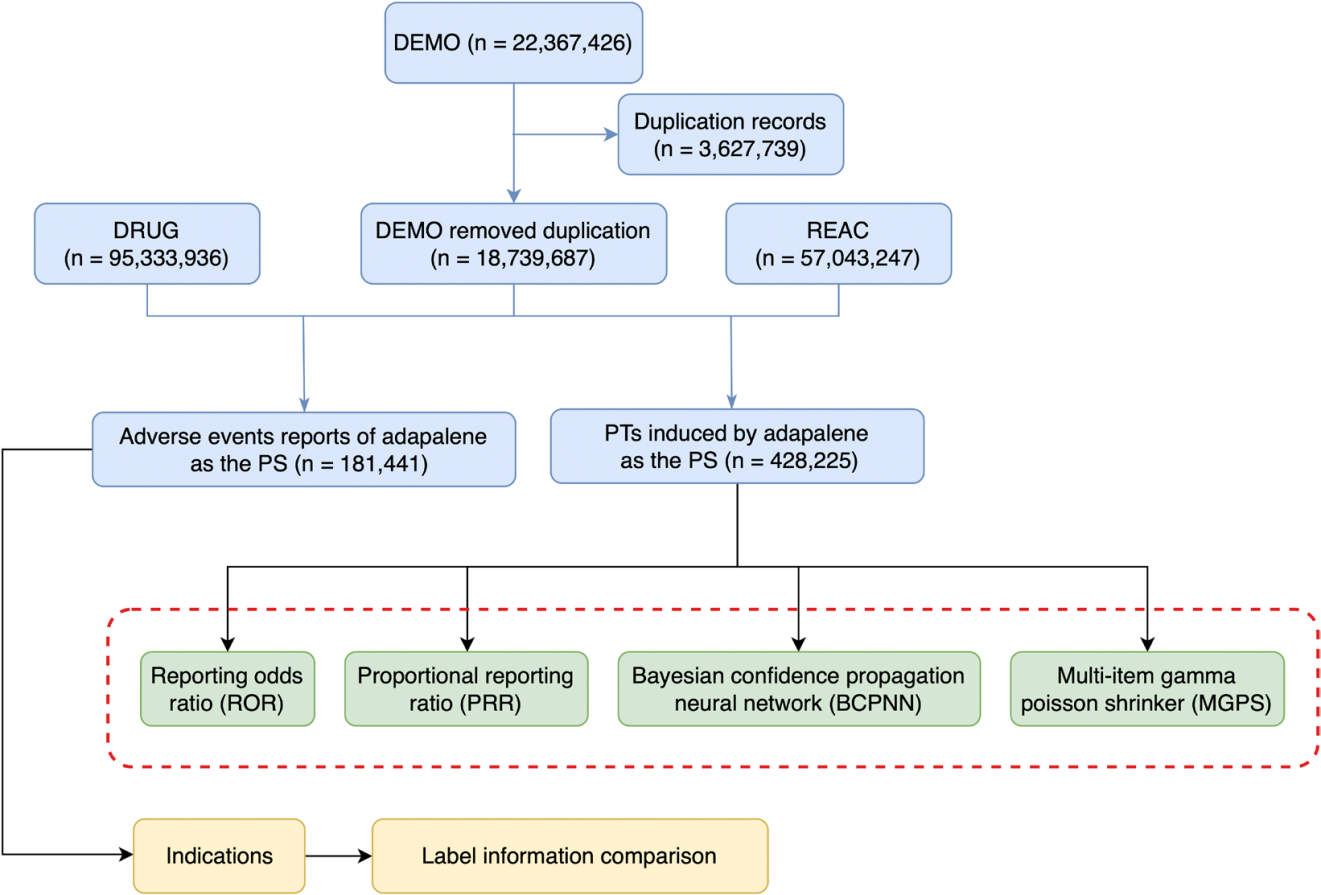
The flow diagram of selecting adapalene‐related AEs from FAERS database.

Skin and subcutaneous tissue disorders accounted for 301 587 records, 70.4% of all adapalene‐associated AE records, and showed the strongest SOC‐level disproportionality (Table S2). Twenty‐two cutaneous PTs satisfied the intersection rule. Skin burning sensation had the highest ROR (234.88; 95% CI, 231.23–238.59), closely followed by skin fragility (232.72; 214.08–252.99). The most frequently reported PTs were dry skin (*n* = 46 237), skin burning sensation (*n* = 42 445), acne (*n* = 40 194), erythema (*n* = 39 137), skin irritation (*n* = 26 625), and skin exfoliation (*n* = 21 980) (Table S3). Signal magnitude alone did not determine clinical interpretation.

Each PT was compared with a frozen U.S. labeling corpus anchored to the December 31, 2025 data cutoff. Adapalene‐only prescribing information and OTC Drug Facts were the primary sources; adapalene/benzoyl peroxide labels provided supportive evidence because fixed combinations were retained. Thirteen PTs were classified as labeled/expected, including burning, irritation, pain, dryness, discoloration, discomfort, exfoliation, erythema, rash, dermatitis, and warmth (Table 1). Acne and acne cystic were classified as indication‐related because they may represent baseline disease, early worsening, progression, or treatment failure. Seven PTs (skin fragility, dilated pores, seborrhoea, sensitive skin, skin swelling, skin tightness, and skin fissures) lacked a defensible monotherapy‐label match and were categorized as potentially under‐recognized. This designation is hypothesis‐generating; it does not establish novelty or causality.

**TABLE 1:** PTs‐level comparison with adapalene labeling and final interpretive classification.

PT	Classification	Code	Label comparison and adjudication basis
Skin burning sensation	Labeled	E1; C	Burning/stinging; skin burning sensation. Exact or near‐exact local tolerability concept
Skin irritation	Labeled	E1; C	Local cutaneous reactions; skin irritation. Explicit label concept
Pain of skin	Labeled	E1/E2	Skin pain; application‐site pain. Direct or clinically equivalent pain concept
Dry skin	Labeled	E1; C	Dryness; dry skin. Exact label concept
Skin hypopigmentation	Labeled	E3; C	Skin discoloration in the monotherapy label, with exact combination‐label support
Skin hyperpigmentation	Labeled	E3; C	Skin discoloration in the monotherapy label, with exact combination‐label support
Skin discomfort	Labeled	E1	Skin discomfort. Exact label concept
Skin exfoliation	Labeled	E2	Desquamation; peeling/scaling. Clinically equivalent local‐reaction concept
Erythema	Labeled	E1; C	Erythema/redness. Exact label concept
Rash papular	Labeled	E3	Rash. The specific papular morphology is nested within the broader labeled concept
Rash macular	Labeled	E3	Rash. The specific macular morphology is nested within the broader labeled concept
Dermatitis	Labeled	E2/E3; C	Contact dermatitis; eczema. Broader PT mapped to explicit label concepts
Skin warm	Labeled	E2	Mild transitory sensation of warmth. Directly supported warmth concept
Acne	Indication‐related	I	Acne vulgaris; early acne worsening. Treated disease/expected early course; indication bias takes precedence
Acne cystic	Indication‐related	I	Acne phenotype. May represent baseline disease, progression, or treatment failure
Skin fragility	Under‐recognized	E4	No defensible monotherapy‐label match; not equivalent to dryness, peeling, or irritation without case‐level evidence
Dilated pores	Under‐recognized	E4	No explicit or clinically equivalent monotherapy‐label concept
Seborrhoea	Under‐recognized	E4	No monotherapy‐label match; not equivalent to dryness or acne
Sensitive skin	Under‐recognized	E4	Photosensitivity and irritation were not treated as equivalent to this nonspecific PT
Skin swelling	Under‐recognized	E4	Only site‐specific face/eye/lip swelling was labeled; the site‐unspecified PT was not equated with allergic edema
Skin tightness	Under‐recognized	E4	No monotherapy‐label match; not equated with dryness or irritation without case detail
Skin fissures	Under‐recognized	E4	Fissuring may follow xerosis, but it is not explicitly represented and was not inferred

*Note:* Primary evidence was the frozen adapalene‐only DailyMed label corpus; combination labels were supportive. Detailed sources and sections are provided in Tables S4 and S5.

Abbreviations: C, additional/supportive combination‐label evidence; E1, exact/verbatim concept; E2, recognized synonym or clinically equivalent concept; E3, broader/narrower concept requiring documented judgment; E4, no defensible monotherapy‐label match; I, indication bias; PT, preferred term.

These results should be interpreted as reporting signals rather than measures of incidence, absolute risk, or severity. FAERS is subject to underreporting, stimulated reporting, missing clinical detail, confounding by indication and co‐medication, and the absence of a denominator for exposed patients. Disproportionality also cannot determine whether adapalene caused an event, and ingredient‐level inclusion of combination products may complicate attribution. Nevertheless, the convergence of four signal‐detection methods and a prespecified label‐adjudication framework helps separate established retinoid tolerability effects from findings that merit case‐level review. Clinicians can use these results to reinforce appropriate application, moisturization, and monitoring for persistent or atypical symptoms without implying that every signal represents a confirmed adverse reaction. Further studies incorporating exposure data, seriousness, outcomes, time to onset, dechallenge/rechallenge, and monotherapy sensitivity analyses are needed to evaluate the under‐recognized group while preserving adapalene's established role in acne therapy [3].

## Author Contributions

All authors extensively discussed the original manuscript. Z.C. and C.Z. conceptualized and wrote the letter. All authors reviewed and approved the final version of the manuscript.

## Funding

The authors have nothing to report.

## Ethics Statement

The authors have nothing to report.

## Consent

The authors have nothing to report.

## Conflicts of Interest

The authors declare no conflicts of interest.

## Supporting information


**Table S1:** ROR, PRR, BCPNN, and EBGM methods, formulas, and thresholds.
**Table S2:** The signal strength of cutaneous adverse events of adapalene at the SOC level in FAERS database.
**Table S3:** The signal strength of cutaneous adverse events of adapalene ranked by case reports number at the PTs level in FAERS database.
**Table S4:** The signal strength of cutaneous adverse events of adapalene ranked by case reports number at the PTs level in FAERS database.
**Table S5:** The signal strength of cutaneous adverse events of adapalene ranked by case reports number at the PTs level in FAERS database.

## Data Availability

This study utilized data from the publicly accessible U.S. Food and Drug Administration (FDA) Adverse Event Reporting System (FAERS), covering the period from the January 2004 to December 2025. FAERS quarterly data files are released in ASCII or XML format and are available free of charge at https://fis.fda.gov/extensions/FPD‐QDE‐FAERS/FPD‐QDE‐FAERS.html. Adverse events in FAERS are coded using the Medical Dictionary for Regulatory Activities (MedDRA). The authors bear sole responsibility for the interpretation and reporting of the data.
