# Supplementary material for: Adapalene‐Associated Cutaneous Adverse Events: A Disproportionality Analysis of the FAERS Database
Source: J Cosmet Dermatol. 2026 Sep 24;25(10):e71207. doi: 10.1111/jocd.71207 (PMC13612837; doi:10.1111/jocd.71207)
Supplement: Supplementary file 1 [file JOCD-25-e71207-s001.docx]

**Adapalene-Associated Cutaneous Adverse Events: A Disproportionality Analysis of the FAERS Database**

**Methods**

This retrospective pharmacovigilance study was conducted using publicly available data extracted from the U.S. FAERS spanning Q1 2004 to Q4 2025. FAERS constitutes the largest global spontaneous reporting system for post-marketing ADR surveillance, consisting of seven interlinked standardized datasets: demographic characteristics (DEMO), drug information (DRUG), adverse reaction descriptions (REAC), patient outcomes (OUTC), report sources (RPSR), drug treatment periods (THER), and therapeutic indications (INDI). Only reports in which adapalene was coded as the primary suspected (PS) drug were included for subsequent analysis.

Rigorous data cleaning was performed to ensure reliability and comparability. Duplicate records were removed using a hierarchical deduplication strategy: for reports sharing identical CASEIDs, only the entry with the latest FDA receipt date (FDA_DT) was retained; if FDA_DT was also identical, the record with the highest PRIMARYID was preserved. Drug nomenclature was standardized to the generic name adapalene to eliminate inconsistencies arising from brand names, dosage forms, and typographical variations.

Cutaneous adverse events were coded and classified in accordance with the Medical Dictionary for Regulatory Activities (MedDRA, version 26.1) at both the System Organ Class (SOC) and Preferred Term (PT) levels. For each cutaneous adverse event term, a standard 2 × 2 contingency table was constructed to support disproportionality analysis. All statistical procedures and data management operations were implemented using R Studio (version 4.3.1).

Signal-identification rule: A drug-PT pair was considered a positive signal only when all four method-specific criteria were met simultaneously: a>=3 and ROR025>1; a>=3 and PRR025>1; IC025>0; and EB05>2. ROR025 and PRR025 denote the lower bounds of the corresponding two-sided 95% confidence intervals; IC025 and EB05 denote one-sided 95% lower credible bounds.

To distinguish findings that mainly reproduce known adapalene tolerability effects from indication-related reporting and signals meriting further evaluation, all 22 positive PTs in Supplemental Table 3 were compared with a frozen U.S. labeling corpus. The primary temporal reference was the label information in force on or before the FAERS data cutoff (December 31, 2025). Labels first revised after that date were used only as a post-cutoff update check and could not retrospectively convert a signal into an unlabeled finding at the study cutoff.

Adapalene-only U.S. prescribing information and OTC Drug Facts were the primary attribution sources. Adapalene/benzoyl peroxide combination labels were secondary sources because the FAERS ingredient-level extraction retained fixed combinations; a combination-only match was flagged as supportive evidence and was not, by itself, considered proof that the event was labeled for adapalene monotherapy. Label sections reviewed included Indications and Usage, Warnings and Precautions, Adverse Reactions (clinical trials and postmarketing), Drug Facts warnings, and patient information.

**Supplemental Table 1.** ROR, PRR, BCPNN, and EBGM methods, formulas, and thresholds.

| **Method** | **Formula** | **Threshold** |
| --- | --- | --- |
| ROR | $ROR=\frac{(a/c)}{(b/d)}=\frac{ad}{bc}$  $SE(\ln ROR)=\sqrt{\left( \frac{1}{a}+\frac{1}{b}+\frac{1}{c}+\frac{1}{d} \right)}$  $95\% CI=e^{\ln(ROR) \pm1.96\sqrt{\left( \frac{1}{a} + \frac{1}{b} + \frac{1}{c} + \frac{1}{d} \right)}}$ | a >= 3 and 95% CI (lower limit) > 1 |
| PRR | $PRR=\frac{a/(a+b)}{c/(c+d)}$  $SE\left( \ln PRR \right)=\sqrt{\frac{1}{a}-\frac{1}{a+b}+\frac{1}{c}-\frac{1}{c+d}}$  $95\% CI=e^{\ln(PRR) \pm1.96\sqrt{\frac{1}{a} - \frac{1}{a+b} + \frac{1}{c} - \frac{1}{c+d}}}$ | a >= 3 and 95% CI (lower limit) > 1 |
| BCPNN | $IC={log}_{2} \frac{p(x,y)}{p(x)p(y)}={log}_{2} \frac{a(a+b+c+d)}{(a+b)(a+c)}$  $E(IC)={log}_{2} \frac{(a+\gamma_{11})(a+b+c+d+\alpha)(a+b+c+d+\beta)}{(a+b+c+d+\gamma)(a+b+\alpha_{1})(a+c+\beta_{1})}$  $V(IC)=\frac{1}{(\ln2)^{2}}\left\{ \frac{(a+b+c+d)-a+\gamma-\gamma_{11}}{(a+\gamma_{11})(1+a+b+c+d+\gamma)}+\frac{(a+b+c+d)-(a+b)+\alpha-\alpha_{1}}{(a+b+\alpha_{1})(1+a+b+c+d+\alpha)}+\frac{(a+b+c+d)-(a+c)+\beta-\beta_{1}}{(a+c+\beta_{1})(1+a+b+c+d+\beta)} \right\}$  $\gamma=\gamma_{11}\frac{(a+b+c+d+\alpha)(a+b+c+d+\beta)}{(a+b+\alpha_{1})(a+c+\beta_{1})}$  $IC-2SD=E(IC)-2\sqrt{V(IC)}$ | IC025 > 0 |
| EBGM | *Empirical Bayes shrinkage method developed by the U.S. FDA, based on a two-component gamma mixture prior fitted across all drug-event pairs in the database. The raw observed-to-expected ratio is shrunk toward the empirical prior distribution to reduce noise from sparse data.EBGM is the geometric mean of the posterior gamma mixture distribution; EBGM₀₅ is the 5th percentile of the posterior distribution (one-sided 95% lower credible bound). No closed-form solution exists; values are computed numerically* | EBGM05 > 2 |

a = number of reports with Adapalene as the primary suspected drug and the target cutaneous adverse events occurred; b = number of reports with Adapalene as the primary suspected drug but the target cutaneous adverse events did not occur; c = number of reports without Adapalene as the primary suspected drug but the target cutaneous adverse events occurred; d = number of reports without Adapalene as the primary suspected drug and the target cutaneous adverse events did not occur; N = total number of cutaneous adverse events reports included in the analysis. This table presents the core formulas and positive signal thresholds for reporting odds ratio (ROR), proportional reporting ratio (PRR), Bayesian confidence propagation neural network (BCPNN, measured by information component, IC), and multi-item gamma Poisson shrinker (MGPS, measured by empirical Bayes geometric mean, EBGM).

**Supplemental Table 2.** The signal strength of cutaneous adverse events of adapalene at the SOC level in FAERS database.

| **SOC** | **Case Reports** | **ROR**  **(95% CI)** | **PRR**  **(95% CI)** | **χ**^2^ | **IC**  **(IC025)** | **EBGM**  **(EBGM05)** |
| --- | --- | --- | --- | --- | --- | --- |
| Skin and subcutaneous tissue disorders | 301587 | 43.31 (43.02, 43.6) | 13.51 (13.51, 13.51) | 3361845.13 | 3.63 (3.62) | 12.35 (12.28) |
| General disorders and administration site conditions | 62960 | 0.77 (0.77, 0.78) | 0.81 (0.81, 0.81) | 3539.51 | -0.31 (-0.32) | 0.81 (0.8) |
| Injury, poisoning and procedural complications | 47199 | 1.07 (1.06, 1.08) | 1.06 (1.06, 1.06) | 189.2 | 0.09 (0.07) | 1.06 (1.05) |
| Immune system disorders | 8459 | 1.7 (1.67, 1.74) | 1.69 (1.66, 1.72) | 2374.96 | 0.75 (0.72) | 1.68 (1.65) |
| Eye disorders | 4489 | 0.49 (0.47, 0.5) | 0.49 (0.48, 0.5) | 2394.31 | -1.02 (-1.06) | 0.49 (0.48) |
| Nervous system disorders | 1075 | 0.03 (0.02, 0.03) | 0.03 (0.03, 0.03) | 38610.4 | -5.11 (-5.2) | 0.03 (0.03) |
| Infections and infestations | 1043 | 0.04 (0.04, 0.04) | 0.04 (0.04, 0.04) | 23429.04 | -4.52 (-4.61) | 0.04 (0.04) |
| Gastrointestinal disorders | 584 | 0.01 (0.01, 0.02) | 0.02 (0.02, 0.02) | 40904.23 | -6.03 (-6.15) | 0.02 (0.01) |
| Respiratory, thoracic and mediastinal disorders | 306 | 0.01 (0.01, 0.02) | 0.01 (0.01, 0.01) | 21923.83 | -6.12 (-6.28) | 0.01 (0.01) |
| Vascular disorders | 141 | 0.01 (0.01, 0.02) | 0.01 (0.01, 0.01) | 9464.21 | -6.07 (-6.31) | 0.01 (0.01) |
| Psychiatric disorders | 125 | 0 (0, 0.01) | 0.01 (0.01, 0.01) | 26080.84 | -7.62 (-7.88) | 0.01 (0) |
| Investigations | 47 | 0 (0, 0) | 0 (0, 0) | 29078.03 | -9.17 (-9.58) | 0 (0) |
| Congenital, familial and genetic disorders | 36 | 0.03 (0.02, 0.04) | 0.03 (0.02, 0.04) | 1253.18 | -5.19 (-5.65) | 0.03 (0.02) |
| Neoplasms benign, malignant and unspecified (incl cysts and polyps) | 36 | 0 (0, 0) | 0 (0, 0) | 11862.18 | -8.32 (-8.79) | 0 (0) |
| Pregnancy, puerperium and perinatal conditions | 35 | 0.02 (0.01, 0.03) | 0.02 (0.01, 0.03) | 1795.27 | -5.72 (-6.19) | 0.02 (0.01) |
| Musculoskeletal and connective tissue disorders | 35 | 0 (0, 0) | 0 (0, 0) | 25000.3 | -9.39 (-9.86) | 0 (0) |
| Ear and labyrinth disorders | 15 | 0.01 (0, 0.01) | 0.01 (0.01, 0.02) | 1911.85 | -7 (-7.71) | 0.01 (0.01) |
| Cardiac disorders | 14 | 0 (0, 0) | 0 (0, 0) | 11759.97 | -9.67 (-10.4) | 0 (0) |
| Blood and lymphatic system disorders | 12 | 0 (0, 0) | 0 (0, 0) | 7759.01 | -9.3 (-10.09) | 0 (0) |
| Reproductive system and breast disorders | 11 | 0 (0, 0.01) | 0 (0, 0) | 3615.33 | -8.35 (-9.16) | 0 (0) |
| Hepatobiliary disorders | 6 | 0 (0, 0) | 0 (0, 0) | 4159.47 | -9.42 (-10.49) | 0 (0) |
| Metabolism and nutrition disorders | 5 | 0 (0, 0) | 0 (0, 0) | 9795.97 | -10.89 (-12.05) | 0 (0) |

**Supplemental Table 3.** The signal strength of cutaneous adverse events of adapalene ranked by case reports number at the PTs level in FAERS database.

| **SOC** | **PT** | **Case Reports** | **ROR (95% CI)** | **PRR (95% CI)** | **χ^2^** | **IC (IC025)** | **EBGM (EBGM05)** |
| --- | --- | --- | --- | --- | --- | --- | --- |
| Skin and subcutaneous tissue disorders | Skin burning sensation | 42445 | 234.88 (231.23, 238.59) | 211.7 (207.59, 215.89) | 3425930.51 | 6.36 (6.34) | 82 (80.93) |
| Skin and subcutaneous tissue disorders | Skin fragility | 1519 | 232.72 (214.08, 252.99) | 231.9 (214.41, 250.81) | 126809.37 | 6.41(6.31) | 84.84 (79.11) |
| Skin and subcutaneous tissue disorders | Skin irritation | 26625 | 207.21 (203.29, 211.22) | 194.39 (190.62, 198.24) | 2075256.64 | 6.31 (6.29) | 79.29 (78.03) |
| Skin and subcutaneous tissue disorders | Acne | 40194 | 169.62 (167.15, 172.14) | 153.8 (150.81, 156.84) | 2824213.55 | 6.16 (6.14) | 71.63 (70.75) |
| Skin and subcutaneous tissue disorders | Acne cystic | 1520 | 141.52 (131.65, 152.14) | 141.02 (130.39, 152.52) | 102259.3 | 6.1 (6.01) | 68.75 (64.71) |
| Skin and subcutaneous tissue disorders | Dilated pores | 44 | 126.47 (83.66, 191.21) | 126.46 (83.79, 190.86) | 2799.16 | 6.03 (5.51) | 65.12 (46.08) |
| Skin and subcutaneous tissue disorders | Pain of skin | 12706 | 125.65 (122.63, 128.74) | 121.95 (119.58, 124.36) | 793218.25 | 6 (5.97) | 63.92 (62.63) |
| Skin and subcutaneous tissue disorders | Seborrhoea | 1639 | 109.36 (102.43, 116.76) | 108.94 (102.72, 115.54) | 96109.94 | 5.91 (5.83) | 60.18 (56.97) |
| Skin and subcutaneous tissue disorders | Sensitive skin | 2890 | 92.71 (88.4, 97.23) | 92.09 (88.55, 95.77) | 153502.15 | 5.77 (5.71) | 54.69 (52.56) |
| Skin and subcutaneous tissue disorders | Dry skin | 46237 | 83.74 (82.75, 84.74) | 74.81 (73.36, 76.29) | 2155099.41 | 5.59 (5.57) | 48.14 (47.66) |
| Skin and subcutaneous tissue disorders | Skin hypopigmentation | 628 | 79.95 (72.41, 88.28) | 79.83 (72.38, 88.05) | 30482.24 | 5.65 (5.52) | 50.15 (46.16) |
| Skin and subcutaneous tissue disorders | Skin hyperpigmentation | 2833 | 71.48 (68.29, 74.83) | 71.02 (68.29, 73.86) | 127243.86 | 5.54 (5.48) | 46.55 (44.8) |
| Skin and subcutaneous tissue disorders | Skin discomfort | 884 | 67.58 (62.31, 73.28) | 67.44 (62.35, 72.94) | 38318.14 | 5.49 (5.38) | 45 (42.05) |
| Skin and subcutaneous tissue disorders | Skin swelling | 2360 | 54.13 (51.59, 56.78) | 53.83 (51.76, 55.98) | 86972.45 | 5.27 (5.2) | 38.54 (37.03) |
| Skin and subcutaneous tissue disorders | Skin exfoliation | 21980 | 52.16 (51.34, 52.99) | 49.53 (48.57, 50.51) | 761413.44 | 5.18 (5.16) | 36.3 (35.83) |
| Skin and subcutaneous tissue disorders | Skin tightness | 959 | 35.41 (32.98, 38.03) | 35.34 (32.68, 38.22) | 25250.6 | 4.81 (4.71) | 28.09 (26.47) |
| Skin and subcutaneous tissue disorders | Erythema | 39137 | 35.39 (34.99, 35.8) | 32.25 (31.62, 32.89) | 956054.38 | 4.71 (4.69) | 26.12 (25.87) |
| Skin and subcutaneous tissue disorders | Rash papular | 4236 | 32.39 (31.31, 33.5) | 32.08 (30.85, 33.36) | 102670.66 | 4.7 (4.65) | 26.01 (25.28) |
| Skin and subcutaneous tissue disorders | Rash macular | 3988 | 17.89 (17.31, 18.49) | 17.73 (17.05, 18.44) | 55553.16 | 3.98 (3.93) | 15.75 (15.32) |
| Skin and subcutaneous tissue disorders | Skin fissures | 2060 | 16.89 (16.13, 17.69) | 16.82 (16.17, 17.49) | 27195.82 | 3.91 (3.84) | 15.03 (14.47) |
| Skin and subcutaneous tissue disorders | Dermatitis | 2106 | 15.85 (15.15, 16.59) | 15.78 (15.17, 16.41) | 26050.77 | 3.83 (3.76) | 14.2 (13.67) |
| Skin and subcutaneous tissue disorders | Skin warm | 602 | 15.8 (14.52, 17.19) | 15.78 (14.59, 17.07) | 7443.85 | 3.83 (3.71) | 14.2 (13.23) |

**Supplemental Table 4.** The signal strength of cutaneous adverse events of adapalene ranked by case reports number at the PTs level in FAERS database.

| **Product/Formulation** | **DailyMed SETID** | **Label Date Used** | **Role** | **Sections/Concepts Reviewed** |
| --- | --- | --- | --- | --- |
| Adapalene Gel 0.3% (monotherapy, Rx) | 56045eee-e84d-467a-9e12-e62bd474ed8a | Revised Sep 2025 | Primary; at-cutoff | Sections 1, 5.1-5.3, 6.1-6.2, 17; irritation, erythema, scaling, dryness, burning/stinging, pain, peeling, warmth, rash, discoloration, hypersensitivity |
| DIFFERIN Gel 0.1% (monotherapy, OTC) | 0739d631-171b-42a8-bd55-0022b8df2d8a | Updated Dec 7, 2022 | Primary; at-cutoff | Drug Facts and consumer information; redness, itching, dryness, burning, allergic swelling, early acne worsening |
| EPIDUO Gel 0.1%/2.5% (combination) | a0610f87-3b39-458a-afa3-66be1fefd1de | Label in force at cutoff; revised Feb 2018 | Secondary; combination-support flag | Sections 5.2, 6.1-6.2; dryness, contact dermatitis, application-site burning/irritation, erythema, scaling |
| EPIDUO FORTE 0.3%/2.5% (combination) | f4e5d381-098a-4c93-9ff0-002cae46daac | Revised Apr 2022 | Secondary; combination-support flag | Sections 5.3, 6.1-6.2; irritation, eczema/dermatitis, burning, pigment changes, blister, pruritus |
| Adapalene Gel 0.3% and EPIDUO update audit | c448eab6-c054-4c8b-9319-556b7e1be3cf; a0610f87-3b39-458a-afa3-66be1fefd1de | Revised Mar and Jun 2026 | Post-cutoff sensitivity check only | Checked for later label changes; not used to define whether a PT was labeled at the 2025 data cutoff |

**Supplemental Table 5.** The signal strength of cutaneous adverse events of adapalene ranked by case reports number at the PTs level in FAERS database.

| **Step** | **Operation** | **Prespecified Rule** | **Audit Field Retained** |
| --- | --- | --- | --- |
| 1 | Freeze sources | Record product, active ingredient(s), SETID, label revision/update date, access date, and evidentiary role before classification. | Product; SETID; date; primary/secondary role |
| 2 | Extract label concepts | Extract cutaneous concepts from Sections 5, 6, and 17 or OTC Drug Facts; retain clinical-trial, postmarketing, warning, and patient-information wording. | Verbatim label concept and section |
| 3 | Normalize terminology | Map label wording to MedDRA version 26.1 without altering the hierarchy. Use the current LLT/PT relationship, synonyms, and medical judgment consistent with MedDRA Term Selection: Points to Consider. | Mapped PT; MedDRA version |
| 4 | Assign match level | E1=exact PT or verbatim concept; E2=recognized synonym/LLT-parent or clinically equivalent concept; E3=broader/narrower concept requiring documented judgment; E4=no defensible match. C denotes support found only or additionally in a combination label. | E1-E4; optional C flag; rationale |
| 5 | Screen indication bias | Determine whether the PT is the treated disease, a disease phenotype, early worsening, progression, or lack of effectiveness. This assessment is made independently of disproportionality magnitude. | Indication-bias yes/no and reason |
| 6 | Apply category precedence | Indication/disease-related takes precedence for acne phenotypes. Otherwise, a defensible monotherapy-label E1-E3 match is labeled/expected. A PT with E4 for monotherapy and no indication explanation is potentially under-recognized. | Final stratum and precedence rule |
| 7 | Adjudicate | Two authors reviewed every PT and resolved disagreements by consensus. The final matrix preserves the consensus rationale; pre-consensus ratings were not retained, so no kappa statistic is reported. | Reviewer initials; consensus rationale |
| 8 | Interpret conservatively | Potentially under-recognized means absent as a defensible concept from the frozen monotherapy-label corpus. It does not mean novel, causal, severe, or more frequent, and it requires case-level and external confirmation. | Interpretive limitation |

**Notes:** The label comparison was independent of ROR magnitude and report count. Final distribution: labeled/expected, 13 PTs; indication/disease-related, 2 PTs; potentially under-recognized, 7 PTs.
